# [^18^F]FDG-PET/CT metabolic parameters as useful prognostic factors in cervical cancer patients treated with chemo-radiotherapy

**DOI:** 10.1186/s13014-016-0614-x

**Published:** 2016-03-16

**Authors:** Fernanda G. Herrera, Thomas Breuneval, John O. Prior, Jean Bourhis, Mahmut Ozsahin

**Affiliations:** Department of Radiation Oncology, Lausanne University Hospital, Lausanne, Switzerland; Department of Nuclear Medicine and Molecular Imaging, Lausanne University Hospital, Lausanne, Switzerland

**Keywords:** [^18^F]FDG-PET/CT, Cervical cancer, Chemo-radiotherapy, Tumor glycolytic volume, Standard uptake value

## Abstract

**Background:**

To compare the prognostic value of different anatomical and functional metabolic parameters determined using [^18^F]FDG-PET/CT with other clinical and pathological prognostic parameters in cervical cancer (CC).

**Methods:**

Thirty-eight patients treated with standard curative doses of chemo-radiotherapy (CRT) underwent pre- and post-therapy [^18^F]FDG-PET/CT. [^18^F]FDG-PET/CT parameters including mean tumor standardized uptake values (SUV), metabolic tumor volume (MTV) and tumor glycolytic volume (TGV) were measured before the start of CRT. The post-treatment tumor metabolic response was evaluated. These parameters were compared to other clinical prognostic factors. Survival curves were estimated by using the Kaplan-Meier method. Cox regression analysis was performed to determine the independent contribution of each prognostic factor.

**Results:**

After 37 months of median follow-up (range, 12–106), overall survival (OS) was 71 % [95 % confidence interval (CI), 54–88], disease-free survival (DFS) 61 % [95 % CI, 44–78] and loco-regional control (LRC) 76 % [95 % CI, 62–90]. In univariate analyses the [^18^F]FDG-PET/CT parameters unfavorably influencing OS, DFS and LRC were pre-treatment TGV-cutoff ≥562 (37 vs. 76 %, *p* = 0.01; 33 vs. 70 %, *p* = 0.002; and 55 vs. 83 %, *p* = 0.005, respectively), mean pre-treatment tumor SUV cutoff ≥5 (57 vs. 86 %, *p* = 0.03; 36 vs. 88 %, *p* = 0.004; 65 vs. 88 %, *p* = 0.04, respectively) and a partial tumor metabolic response after treatment (9 vs. 29 %, *p* = 0.0008; 0 vs. 83 %, *p* < 0.0001; 22 vs. 96 %, *p* < 0.0001, respectively). After multivariate analyses a partial tumor metabolic response after treatment remained as an independent prognostic factor unfavorably influencing DFS and LRC (RR 1:7.7, *p* < 0.0001, and RR 1:22.6, *p* = 0.0003, respectively) while the pre-treatment TGV-cutoff ≥562 negatively influenced OS and DFS (RR 1:2, *p* = 0.03, and RR 1:2.75, *p* = 0.05).

**Conclusions:**

Parameters capturing the pre-treatment glycolytic volume and metabolic activity of [^18^F]FDG–positive disease provide important prognostic information in patients with CC treated with CRT. The post-therapy [^18^F]FDG-PET/CT uptake (partial tumor metabolic response) is predictive of disease outcome.

## Background

Cervical cancer (CC) is the second most common cancer among women in the world and a leading cause of cancer mortality [1]. Since the publication in 1999 of randomized trials using platinum-based chemotherapy (PBCT) in patients with locally-advanced cervical cancer (LACC) and the clinical recommendations announced by the National Cancer Institute, concurrent radiotherapy (RT) and cisplatin-based chemotherapy became the standard management with a significant improvement in survival rates compared with RT alone [2–6]. More recently other trials suggested that there might be a benefit for sequential chemotherapy after standard concomitant chemo-radiotherapy (CRT) and this therapeutic approach is now being tested in randomized trials [7, 8]. The gains in survival observed with CRT have come at a substantial price because both acute and late morbidities increased with combined modality treatment. It is thus expected that intensifying the PBCT regimen would increase side effects and reduce the patient’s quality of life. Even with the significant reductions in the risk of CC death observed with CRT, the absolute gains are small for patients with early tumors, many of whom could have been cured with RT alone and recurrence rates are still high for patients with very large or advanced tumors for whom adjuvant chemotherapy might be of benefit [7]. It is therefore important to establish accurate predictors of CRT response, particularly if adjuvant chemotherapy after primary CRT should become the new treatment paradigm.

Some clinical and pathological prognostic factors including age, histological tumor type, tumor grade, large tumor size, International Federation of Gynecology and Obstetrics (FIGO) stage, pelvic and para-aortic lymph node metastasis, lympho-vascular space invasion (LVSI) and anemia are currently considered important for survival and/or treatment outcome [9–11]. By using these prognostic factors and despite carefully implemented treatment it is estimated that 30-40 % of patients with LACC will still recur and eventually die from the disease. Therefore additional research efforts have concentrated on prognostic factors to accurately predict the prognoses of patients with LACC [12]. Advances in the understanding of the tumor microenvironment such as hypoxia [11, 13, 14], interstitial fluid pressure [15, 16], angiogenesis [17, 18] and the immune landscape [19, 20] open the window to implement new therapeutic approaches and to tailor intensified treatment to those patients considered as having a significant risk of recurrence or death. There is a high level of evidence that ^18^F-labeled fluorodeoxyglucose positron emission tomography [^18^F]FDG-PET/CT plays an essential role in the primary evaluation of CC, particularly in evaluating lymph nodal status and distant metastases, contributing to precise tumor staging and changes in therapeutic attitudes [21]. [^18^F]FDG-PET/CT is also important as a predictor of treatment outcome after CRT [22]. The maximum standardized uptake value (SUVmax) of the primary tumor, a semi-quantitative parameter derived from [^18^F]FDG-PET/CT, is known to be a significant prognostic factor in many types of cancers as well as CC [23–25]. However, this parameter is a single voxel value and does not reflect the metabolism of the whole tumor. Other volumetric [^18^F]FDG-PET/CT parameters such as average SUV (SUVmean), metabolic tumor volume (MTV) and total glycolytic activity within the tumor volume (TGV) are easily acquired using a threshold-based automatic volume of interest technique [26].

In this study we compared the prognostic values of various volume-based metabolic parameters including SUVmax, SUVmean, MTV, and TGV determined using [^18^F]FDG-PET/CT with other clinical and pathological prognostic parameters in patients with LACC treated with CRT. The purpose of this study was to establish whether tumor SUV, MTV and TGV add prognostic information to clinical prognostic factors in patients with LACC.

## Methods

After approval by the local ethics committee, we retrospectively reviewed the charts of 38 consecutive eligible patients who presented an intact LACC and were treated with concomitant cisplatin and RT between 2007 and 2014. Inclusion criteria were pathological diagnosis of CC, stage IB1-IVA according to the FIGO 2009 definition. Patients with stage IB1 and IIA1 were considered for inclusion if they had positive lymph nodes. Patients were also required to have staging by examination under anesthesia, [^18^F]FDG-PET/CT imaging and pelvic magnetic resonance imaging (MRI) at diagnosis, a minimum 6-month follow-up and a second [^18^F]FDG-PET/CT image three months after completing CRT. Exclusion criteria were history of previous chemotherapy or RT and metastatic disease. Patients with positive pelvic/para-aortic lymph nodes were not considered as FIGO IVB (metastatic) because these patients can still undergo a curative cisplatin-based CRT treatment. We thus included patients with positive nodes in our analysis.

Data were obtained from the electronic and written medical records and included age at diagnosis, date of diagnosis, histology, grade, presence of LVSI, FIGO stage, presence of positive lymph nodes confirmed by [^18^F]FDG-PET/CT, tumor size measured on MRI, tumor SUVmean, tumor SUVmax, MTV, TGV, body mass index, complete blood counts (CBC) before treatment (white blood cells, platelets, hemoglobin), RT dose (including brachytherapy dose), metabolic tumor response measured by [^18^F]FDG-PET/CT at three months post-treatment, SUVmax, SUVmean, TGV and MTV of tumors at recurrence, date and location of recurrence, date of last follow-up and date of death.

Recurrences were grouped into local (vaginal and/or cervical recurrences), regional (pelvic/para-aortic recurrences), and distant (upper abdominal and/or extra-abdominal).

### [^18^F]FDG-PET/CT acquisition parameters

All patients fasted for at least 6 h before the PET study. The [^18^F]FDG-PET/CT was performed 60 min after administration of 3.5 MBq/kg [^18^F]]FDG (Discovery 690FX TOF; GE Healthcare, Milwaukee, WI) and images (from the head to proximal thigh) were reconstructed using time-of-flight and point-spread function information. The SUV was calculated by correcting for the injected dose of [^18^F]-FDG and patient’s body weight. An increase in FDG uptake above a SUV of 2.5 g/ml was used to define malignancy based on previous publications [27–29].

### [^18^F]FDG-PET-CT tumor measurements

All PET/CT studies were retrieved from the electronic archival system and were then reviewed on a workstation (Velocity Advanced Image Software; Velocity Medical Solutions, Atlanta, GA), PET, CT, and deformable registered PET/CT images were reviewed in axial, coronal and sagittal planes. For the purposes of this study the relevant imaging biomarker measurements were tumor SUVmax, SUVmean, MTV and TGV obtained from the pre-treatment PET. The SUVmax was defined as the maximum SUV within the tumor. SUVmean was calculated by computing the average SUV within a region of interest (tumor). The MTV was defined as the FDG avid tumor volume measured bi-dimensionally at the longest diameter. The total glycolytic activity within the tumor volume or TGV was defined as follows:

$$ \mathrm{M}\mathrm{T}\mathrm{V}\kern0.5em \times \kern0.5em \mathrm{SUVmean}, $$

where MTV is the metabolic tumor volume and SUVmean is the mean SUV. Once the primary tumor was auto-segmented, the software automatically calculated the SUVmax, SUVmean, MTV, and TGV [26].

Metabolic tumor volume defines the volume of the tumor on the basis of the distribution of metabolic activity instead of the traditional CT densities that depend on electron density of the target. TGV goes a step further and effectively weighs this volume by its mean metabolic activity. Hence, a large TGV may reflect a small volume with high metabolic activity (high SUVmean) or a large volume with a lower metabolic activity. These two conditions are different and may be related to different tumor blood supply, microenvironment conditions, tumor hypoxia, etc. [30]. To better assess the outcome of these patients, taking into account these two different conditions, we dichotomized the MTV and the SUVmean at the sample median. This dichotomization allowed us to group patients below or above the variable in question (group A: patients with MTV below the median, group B: patients with MTV above the median, group C: patients with SUV below the median, group D: patients with SUV above the median). Based on the MTV and SUV dichotomization, patients were then stratified in four different categories:

- Group A + C: Patients with MTV below the median + patients with mean SUV below the median
- Group A + D: Patients with MTV below the median + patients with mean SUV above the median
- Group B + C: Patients with MTV above the median + patients with mean SUV below the median
- Group B + D: Patients with MTV above the median + patients with mean SUV above the median

Post-treatment metabolic response was determined qualitatively using the following definitions: complete metabolic response (CMR) was defined as absence of abnormal FDG uptake at sites of abnormal FDG uptake noted on the pre-treatment [^18^F]FDG-PET/CT study. Partial metabolic response (PMR) was defined as any persistent abnormal FDG uptake at these sites. Progressive disease (PD) was defined as any new sites of abnormal FDG uptake that were not present on the pre-treatment PET [22].

We also obtained the SUVmax, SUVmean, MTV, and TGV from the [^18^F]FDG-PET/CT from patients that had partial metabolic response after chemo-radiotherapy treatment.

### Cisplatin and radiotherapy administration

Chemotherapy consisted of weekly cisplatin (40 mg/m^2^) delivered concurrently with RT. Neither induction nor adjuvant chemotherapy was administered.

Before starting RT treatment, patients underwent a pelvic computed tomography (CT) scan (planning scan) with intravenous contrast medium. They were scanned in a supine position with a head and knee support. For the CT-planning scan, standard acquisition parameters were used (tension, 120 kV; tube rotation time 1 s; tube current, 160 mA; helical acquisition with pitch of 0.938; reconstructed image thickness, 2 mm). Consortium guidelines were used to contour the pelvic clinical target volume (CTV) [31]. According to the same guidelines, a 1.5-cm uniform margin was added around the CTV to obtain the pelvic planning target volume (PTV). The nodal CTV was delineated according to the published guidelines, with a 7-mm uniform margin to obtain the nodal PTV [32]. The small bowel, sigmoid, rectum, bladder and femoral heads were contoured as organs at risk. Treatment plans were performed using the Tomotherapy treatment planning system (Accuray Inc, Sunnyvale, CA) with a field width of 5 cm and a pitch of 0.287. Pelvic radiation dose was 45–50.4 Gy in 1.8-Gy daily fractions, in 25–28 fractions. In patients with positive pelvic or para-aortic nodes, extended field RT was used to a dose of 45 Gy with a simultaneous integrated boost to the positive nodes of 60 Gy in 2.4-Gy per fraction in 25 fractions. Daily image guidance before each fraction was implemented using the MV fan-beam CT of Tomotherapy. Patients received 3–4 fractions of MRI-guided high-dose-rate (HDR) intracavitary brachytherapy every 4 days. The prescribed dose was 7 Gy to the high-risk CTV. Brachytherapy was administered after the end of external beam RT.

### Clinical and imaging follow-ups

Clinical follow-up exams of the patients were performed weekly during CRT and after the completion of therapy as follows: every 3 months until 24 months, every 6 months during years 2–5, and annually thereafter. All the patients in this series had a [^18^F]FDG-PET/CT evaluation at three months following CRT to assess tumor response. Subsequently follow-up imaging studies consisted of MRI/CT and/or [^18^F]FDG-PET/CT if clinically indicated. Recurrences were biopsy/surgical specimen proven in more than 50 % of the cases of recurrent disease and in the remainder of the cases recurrences were proved by [^18^F]FDG-PET/CT.

### Statistical considerations

Survival curves were computed using the Kaplan-Meier method [33]. Time to any event was measured from the day of diagnosis. Death certificates confirmed date of deaths. If clinical or pathological evidence of active, recurrent disease was present, deaths were attributed to CC. The events were death (all causes) for overall survival (OS) and death (all causes) or relapse for disease-free survival (DFS). For the loco-regional control (LRC) rate the event consisted of local or regional relapse.

Confidence intervals (CI) were calculated from standard errors. In univariate analyses differences between groups were assessed using the log-rank test.

All variables were dichotomized using cutoffs reported in the literature when available or the best discriminating value as determined by recursive partitioning [34].

In multivariate analyses we screened for prognostic factors with a *p*-value of less than 0.05 in univariate analyses by using the Cox regression analysis to define the independent contribution of each prognostic factor [35].

Unpaired Student’s *t* test was used to compare means of two individual groups.

A *p* value of **≤** 0.05 was considered to be statistically significant. All data were examined using JMP version 10.0.0 (SAS Institute Inc., Cary, NC).

## Results

### Patient and tumor characteristics

A total of 38 patients with LACC were identified who fulfill the inclusion criteria. Patients’ surgical, pathological and treatment characteristics are detailed in Table 1. The median age was 52.5 years (range, 26–83 years). The median tumor size was 4.5 cm (range, 2–8 cm). The median body-mass index at diagnosis was 23 (range, 14–42). The median CBC at baseline was 8.55 · 10^3^/mL (range, 4.6–25.6) for white blood cells; 130 g/dL (range, 71–149) for hemoglobin; 273.5 · 10^3^/mL (range, 186–819) for platelets.

**Table 1:** Patient and tumor characteristics in 38 patients treated with cervical cancer treated with chemo-radiotherapy

FIGO stage	*N* = 38	Percent
IB1	2	5.2
IB2	4	10.5
IIA1	6	15.7
IIA2	4	10.5
IIB	15	39.5
IIIA	2	5.2
IIIB	4	10.5
IVA	1	2.6
Histology		
Squamous-cell carcinoma	33	87
Adenocarcinoma	5	13
Grade		
1	8	21
2	23	60.5
3	7	18.5
Lymph nodal status		
Pelvic positive	10	26.3
Pelvic and para-aortic positive	12	31.6
Negative lymph nodes	16	42.1
Lymphovascular space invasion		
Positive	26	68.4
Negative	12	31.5

The pre-treatment tumors’ average SUVmax, SUVmean, MTV, and TGV (range) were 17.8 g/mL (4.3–34.6), 4.24 g/mL (2–8.28), 114.16 cm^3^ (19.73–546.12), and 493.75 (55.83–2991); respectively.

In patients with PMR, the three-months post-treatment primary tumor’s average SUVmax, SUVmean, MTV, and TGV (range) were 18.4 g/mL (4–20), 10.38 g/mL (3.5–17), 43.15 cm^3^ (6–35.4), 521.64 (72–2496); respectively.

### Cisplatin and radiotherapy treatment

All patients received, as planned, 5 cycles of cisplatin combined with RT. Four patients had cisplatin dose modifications: 2 because of renal toxicity and 2 because of leucopenia. In these 4 patients the cisplatin dose was reduced to 30 mg/m^2^ and 20 mg/m^2^ respectively. The median pelvic radiation dose was 45 Gy (range, 45–50.4 Gy). Twenty-two patients received extended-field RT to the para-aortic lymph nodes. All women received HDR brachytherapy with a median dose of 28 Gy (range, 21–28 Gy) in 3–4 fractions. No patient experienced delays or breaks in RT because of short-term toxicity (median RT duration, 41 days; range, 32–51 days).

### Disease outcome

After a median follow-up period of 37 months (range, 6–106 months), the 3-year OS was 71 % (95 % CI, 54–88), DFS 61 % (95 % CI, 44–78) and LRC 76 % (95 % CI, 62–90). By the end of follow-up 24 out of 38 (63.15 %) patients were alive and without disease. A total of 14 patients (36.84 %) experienced a recurrence. Nine patients had a local recurrence, 3 of them presented also with regional nodal recurrence and five patients had distant metastases. All the patients with recurrence died of CC. The nine patients with local recurrence had a PMR three months post-primary CRT treatment. For these patients the [^18^F]FDG-PET/CT allowed the detection of “in field” local/regional recurrence which were biopsy proven. Five of these patients were offered salvaged surgery, and had a median time since the diagnosis of recurrence to death of 10.5 months being systemic disease the main cause of death. The remaining 4 patients with local PMR presented also systemic disease at the post-treatment [^18^F]FDG-PET/CT and the median time since the diagnosis of recurrence to death was 6 months.

For the nine patients with PMR three months after CRT, the mean (±SD) pre-post-therapy TGV were 957 (±1049) and 521 (±773), respectively (unpaired student *t* test, *p* = 0.33), and the mean (±SD) pre-post-therapy MTV were 126 (±109) and 43 (±46), respectively (unpaired student *t* test, *p* = 0.05).

**Fig. 1:**
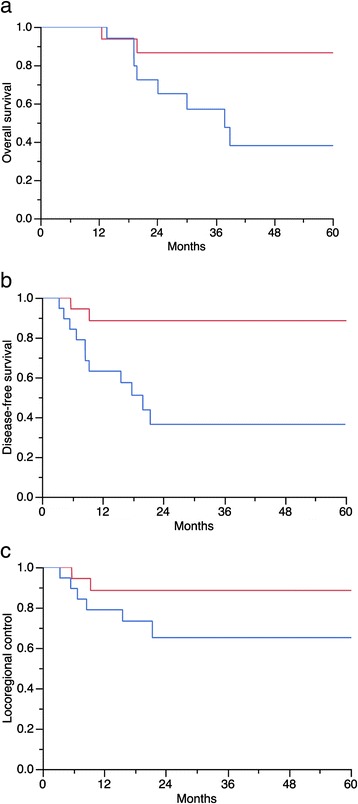
Univariate analysis. [^18^F]FDG-PET/CT Standard uptake value (SUV) mean cutoff ≥5 (*blue curve*) before starting chemo-radiotherapy unfavorably influenced: **a**) Overall survival (86 *vs*. 57 %, *p* = 0.03), **b**) Disease-free survival (88 *vs*. 36 %, *p* = 0.004), and **c**) Locoregional control (88 *vs*. 65 %, *p* = 0.04)

**Fig. 2:**
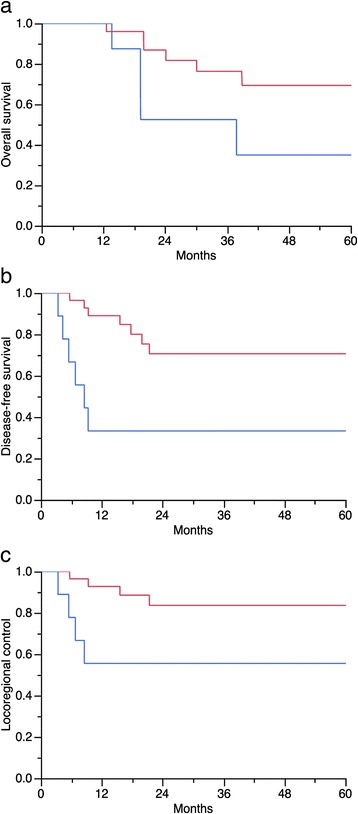
Univariate analysis. [^18^F]FDG-PET/CT tumor glycolytic volume (cutoff ≥562) before starting chemo-radiotherapy unfavorably (*blue curve*) influenced: **a**) Overall survival (37 *vs*. 76 %, *p* = 0.01), **b**) Disease-free survival (33 *vs*. 70 %, *p* = 0.002), and **c**) Locoregional control (55 *vs*. 83 %, *p* = 0.04)

**Fig. 3:**
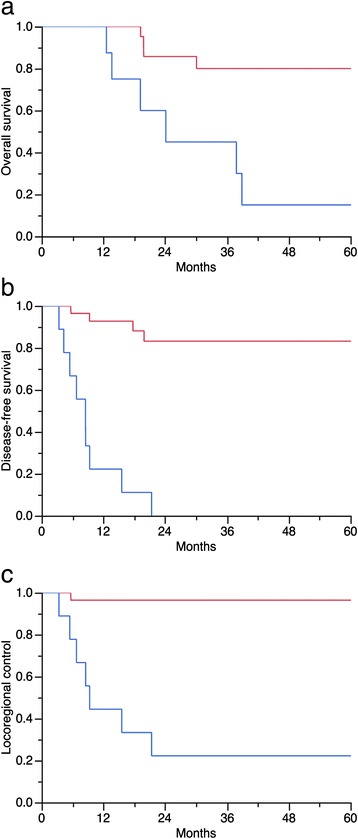
Univariate analysis. [^18^F]FDG-PET/CT partial tumor metabolic response after treatment unfavorable (*blue curve*) influenced: **a**) Overall survival (9 *vs*. 29 %, *p* = 0.0008); **b**) Disease-free survival (0 *vs*. 83 %, *p* < 0.0001); and **c**) Locoregional control (22 *vs*. 96 %, *p* < 0.0001)

**Fig. 4:**
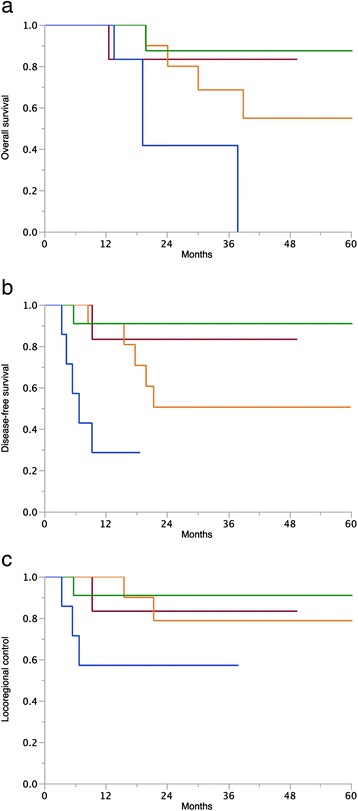
Univariate analysis. Subgroup analysis based on the dichotomization of MTV and SUVmean at the sample median. In green group B + C: Patients with MTV above the median + patients with mean SUV below the median. In red group A + C: Patients with MTV below the median + patients with mean SUV below the median. In orange group A + D: Patients with MTV below the median + patients with mean SUV above the median. In blue group B + D: Patients with MTV above the median + patients with mean SUV above the median. **a**) Overall survival: B + C 87 %, A + C 83 %, A + D 68 %, B + D 0 %; *p* < 0.003. **b**) Disease-free survival: B + C 90 %, A + C 83 %, A + D 50 %, B + D 25 %; *p* < 0.0003. **c**) Locoregional control: B + C 90 %, A + C 83 %, A + D 78 %, B + D 50 %; *p* < 0.03

In univariate analyses (Table 2) the [^18^F]FDG-PET/CT parameters unfavorably influencing OS, DFS and LRC were pre-treatment TGV-cutoff ≥562 (37 *vs*. 76 %, *p* = 0.01; 33 *vs*. 70 %, *p* = 0.002; and 55 *vs*. 83 %, *p* = 0.005, respectively), pre-treatment tumor SUVmean-cutoff ≥5 (57 *vs*. 86 %, *p* = 0.03; 36 *vs*. 88 %, *p* = 0.004; 65 *vs*. 88 %, *p* = 0.04, respectively), and a partial tumor metabolic response after treatment (9 *vs*. 29 %, *p* = 0.0008; 0 *vs*. 83 %, *p* < 0.0001; 22 *vs*. 96 %, *p* < 0.0001, respectively). (Figures 1, 2 and 3). After subgroup analysis based on the MTV and SUV dichotomization, patient’s in-group A + C and B + C had significantly better OS, DFS, and LRC than patient’s in-group A + D and B + D (Table 2 and Fig. 4).

**Table 2:** Univariate analysis in 38 patients treated with cervical cancer treated with chemo-radiotherapy

Variable	*n*	3-y OS (%)	95 % CI	*p*	3-y DFS (%)	95 % CI	*p*	3-y LRC (%)	95 % CI	*p*
All patients	38	71	54–88	-	61	44–78	-	76	62–90	-
Age (years)										
<50	22	41	27–49		63	61–65		72	69–75	
				0.35			0.7			0.85
>50	16	78	74–82		53	50–56		77	60–94	
Tumor size										
≥4.5 cm	15	63	61–65		57	55–59		65	63–67	
				0.5			0.56			0.19
<4.5 cm	23	82	80–84		68	65–71		84	68–100	
Stage FIGO 2009										
IB1, IIA1, IB2	8	71	68–74		57	55–59		71	67–75	
IIA2, IIB	23	74	69–79	0.9	62	60–64	0.84	77	60–94	0.84
IIIA, IIIB, IVA	7	60	56–64		60	56–64		75	71–79	
Histology										
SCC	33	75	58–92		65	48–82	0.02*	79	65–93	
				0.02*						0.15
Adenocarcinoma	5	50	45–55		26	21–31		50	45–55	
LVSI										
Positive	26	63	60–66		58	56–60		72	53–91	
				0.87			0.62			0.55
Negative	12	65	62–68		66	63–69		83	81–85	
Grade										
1	3	66	61–71		66	61–71		66	61–71	
2	18	56	54–58	0.81	53	52–55	0.92	85	71–99	0.33
3	17	84	82–86		69	67–71		69	67–71	
Lymph nodes										
Positive	22	55	52–58		60	57–63		76	58–94	
				0.44			0.66			0.79
Negative	16	74	72–76		62	60–62		76	74–78	
Hemoglobin (g/dl)										
≥100	34	72	55–89		64	47–81		77	63–91	
				0.6			0.1			0.46
<100	4	50	43–57		66	61–71		66	61–71	
WBC (x10^3^/ml)										
≥8.5	19	66	63–69		61	59–63		73	71–75	
				0.95			0.83			0.44
<8.5	19	65	62–67		61	59–63		79	77–81	
Median TGV (cm3)										
≥255	19	59	57–61		52	50–54		69	66–72	
				0.2			0.25			0.38
<255	19	79	77–81		70	68–72		82	65–99	
TGV interquartile range (cm3)										
≥562	29	37	27–43		33	30–36		55	52–58	
				0.01*			0.002*			0.005*
<562	9	76	58–94		70	52–88		83	68–98	
Tumor mean SUV (g/ml)										
≥5	19	57	54–59		36	33–39		65	63–68	
				0.03*			0.004*			0.04*
<5	19	86	72–100		88	70–100		88	78–98	
Mean tumor SUVmax (g/ml)										
≥15	14	61	55–65		53	45–58		62	58–69	
				0.78			0.51			0.09
<15	24	78	73–83		66	60–72		85	75–90	
Three-month post-treatment PET metabolic response										
CMR	29	80	78–82		83			96	93–99	
				0.0008*		68–98	<0.0001*			<0.0001*
PMR	9	30	27–33		0			22	19–25	
MTV										
≥69 cc	19	58	35–39		63	61–65		73	71–75	
				0.65			0.47			0.35
<69 cc	19	73	55–61		63	61–65		81	62–100	
Group										
A + C	8	83	53–100		83	53–100		83	54–100	
B + C	11	87	64–100	0.003*	90	73–100	0.0003*	90	73–100	0.03*
A + D	11	68	38–98		50	19–81		78	52–100	
B + D	8	0	0		25	0–55		50	16–84	

Table abbreviations:

*OS* Overall survival, *DFS* Disease-free survival, *LRC* Loco-regional control, *CI* Confidence interval, *FIGO* International Federation of Gynecology and Obstetrics staging system, *TGV* Total glycolytic activity within the tumor volume, *SUV* Standardized uptake value, *SUVmax* Maximum SUV, *MTV* Metabolic tumor volume, *SCC* Squamous cell carcinoma, *LVSI* Lympho-vascular space invasion, *WBC* White blood cells, *CMR* Complete metabolic response, *PMR* Partial metabolic response

Group categories: Group A + C: Patients with MTV below the median + patients with mean SUV below the median. Group A + D: Patients with MTV below the median + patients with mean SUV above the median. Group B + C: Patients with MTV above the median + patients with mean SUV below the median. Group B + D: Patients with MTV above the median + patients with mean SUV above the median

*p-values statistically significant

Patients with squamous-cell carcinoma had a statistically significant better clinical outcome than patients with adenocarcinomas (OS: 75 vs. 50 %, *p* = 0.02, DFS 65 vs. 26 %, *p* = 0.02).

After multivariate analyses a partial tumor metabolic response after treatment remained as an independent prognostic factor unfavorably influencing DFS and LRC (RR 1:7.7, *p* < 0.0001, and RR 1:22.6, *p* = 0.0003, respectively) while the pre-treatment TGV-cutoff ≥562 negatively influenced OS and DFS (RR 1:2, *p* = 0.03, and RR 1:2.75, *p* = 0.05) (Table 3).

**Table 3:** Cox multivariate analysis in 38 patients with cervical cancer treated with chemo-radiotherapy

Variable	OS		DFS		LRC	
	RR	*p*	RR	*p*	RR	*p*
Three-month post-treatment partial metabolic response by [^18^F]FDG-PET/CT	1:1.5	0.08	1:7.7	<0.0001*	1:22.6	0.0003*
Pre-treatment TGV (cutoff >562 cm^3^)	1:2	0.03*	1:2.75	0.05*	1:3.3	0.07

*OS* Overall survival, *DFS* Disease-free survival, *LRC* Loco-regional control, *RR* Relative risk, *[* ^*18*^ *F]FDG-PET* ^18^F-labeled fluorodeoxyglucose positron emission tomography, *CT* Computed tomography, *TGV* Total glycolytic activity within the tumor volume

*p-value statistically significant

## Discussion

We investigated the prognostic values of [^18^F]FDG-PET/CT volume-based metabolic parameters and we compared them with other clinical and pathological prognostic factors in LACC patients undergoing CRT. Our study shows that the pre-treatment TGV, the pre-treatment tumor SUVmean, and the metabolic response are important independent prognostic factors for survival and recurrence. There is an increasing interest in the utilization of functional parameters in tumor-volume delineation because metabolic data may significantly improve the determination of the biologically relevant volume and the heterogeneity within the tumor. In our series TGV remained statistically significant as a predictor of DFS in the multivariate analysis. It is thus important to differentiate the TGV from other metabolic parameters evaluated in our study (pre-treatment SUVmax, SUVmean and MTV). While standardized uptake value can only represent metabolic activity as a semiquantitative index for tumor uptake, MTV represents the number of active metabolic tumor cells and the combination of both (TGV) is a more effective prognostic factor that takes into account both metabolic activity and tumor volume as important parameters of tumor response to therapy. Our results suggest also that the combination of uptake intensity (SUVmean) and MTV analysis improved the identification of poor prognosis subtype. It is possible that at a local level, these tumor differences of intensity between contiguous voxels represent heterogeneity parameters associated with tumor regions of increased or reduced metabolism determined by different microenvironmental conditions.

The TGV has also been associated with therapeutic response and survival in other tumors [36–38]. The TGV cutoff reported in our series probably reflects the increased recurrence and mortality in patients with large growing tumor burden (metabolically active fraction). For these patients it is probable that the volume of the tumor at presentation determines the mortality within one or two years, linking tumor aggressiveness with disease burden. These patients could be those that benefit from adjuvant intensified PBCT regimens.

Other functional measurements like SUVmean and SUVmax have been reported as prognostic in CC outcome in a number of studies. Kidd et al. showed that the SUVmax of the tumor is a sensitive biomarker for prognosis in patients with uterine CC [23]. In a study where repetitive PET/CT images of CC patients were taken during CRT, the SUVmax shows a consistent rate of decline during treatment and declines at a faster rate than MTV regresses, representing also an earlier tool for prediction of tumor response [39].

One third of patients with LACC will have disease recurrence, usually within 2 years of completing treatment. The most important predictors of disease recurrence include clinical stage, lymph node status at diagnosis, tumor histology and early tumor response after treatment [12, 22]. After CRT as definitive treatment of LACC there is sufficient evidence to support the use of [^18^F]FDG-PET/CT for the assessment of treatment response. The presence of FDG activity (either persistent or new) can predict survival outcome. In accordance with our series, a study in which [^18^F]FDG-PET/CT was performed 3 months after completion of treatment showed that a metabolic response was predictive of long-term survival, with a 3-year survival rate of 78 % in patients with a CMR, 33 % in patients with a PMR and 0 % in those with PD. Multivariate analysis in that study showed that post-treatment response and lymph node status at diagnosis were the only accurate predictors of DFS [22]. Standard surveillance programs have proposed the use of routine physical examinations and patient’s symptoms education to facilitate early disease recurrence. However, by fully exploiting the diagnostic information derived from pre- and post-treatment [^18^F]FDG-PET/CT, patients with adverse metabolic prognostic factors could potentially benefit from either adjuvant systemic therapy or salvage curative therapy in the case of disease recurrence [40, 41]. In our study we observed that in patients with local PMR three-months post CRT, the difference in pre-post treatment mean TGV were not statistically significant (*p* = 0.33), while the pre-post treatment mean MTV was significantly reduced after CRT (*p* = 0.05). This highlights the fact that even if the tumor metabolic volume reduces its size after CRT, the persistent high metabolic activity within the tumor may be responsible for tumor recurrences. This raises the question whether dose escalation strategies are more likely to provide a therapeutic gain.

This study had several limitations including its retrospective design and a relatively small sample size of 38 patients as well as the long accrual period with consequent variations in treatment protocols according to each patient’s situation and corresponding physician’s decision, which might have affected the outcome of the patients. We used the segmentation algorithm from only one commercially available software workstation for image analysis and need to investigate other segmentation algorithms. Despite this, our study provides proof-of-concept to support the clinical value of volumetric functional assessment. Validation in a prospective and larger cohort of patients is warranted.

## Conclusions

In the current study we demonstrated that TGV is one of the most effective independent prognostic parameters for disease outcome in LACC patients undergoing CRT. These findings suggest that combined assessment of the metabolic activity and volume of the primary tumor is more effective for prediction of prognosis in CC than a single parameter alone. These data need to be validated in a larger cohort of patients. We confirmed that the post-treatment partial tumor metabolic response is predictive of disease outcome, and this could have significant implications for the use of adjuvant as well as salvage therapies in LACC patients treated with definitive CRT.

## Abbreviations

[^18^F]FDG-PET: ^18^F-labeled fluorodeoxyglucose positron emission tomography
CBC: Complete blood count
CC: Cervical cancer
CI: Confidence interval
CMR: Complete metabolic response
CRT: Chemo-radiotherapy
CT: Computer tomography
CTV: Clinical target volume
DFS: Disease-free survival
FIGO: International Federation of Gynecology and Obstetrics
HDR: High-dose rate
LACC: Locally-advanced cervical cancer
LRC: Loco-regional control
LVSI: Lympho-vascular space invasion
MRI: Magnetic resonance imaging
MTV: metabolic tumor volume
OS: Overall survival
PBCT: Platinum-based chemotherapy
PD: Progressive disease
PMR: Partial metabolic response
PTV: Planning target volume
RR: Relative risk
RT: Radiotherapy
SUV: Standardized uptake value
SUVmax: Maximum standardized uptake value
SUVmean: Mean standardized uptake value
TGV: Total glycolytic activity within the tumor volume
SD: Standard deviation
